# Morphological descriptions and morphometric discriminant function analysis reveal an additional four groups of *Scylla* spp

**DOI:** 10.7717/peerj.8066

**Published:** 2020-01-03

**Authors:** Hanafiah Fazhan, Khor Waiho, Emilia Quinitio, Juliana C. Baylon, Yushinta Fujaya, Nita Rukminasari, Mohammad Farhan Darin Azri, Md. Sheriff Shahreza, Hongyu Ma, Mhd Ikhwanuddin

**Affiliations:** 1 Guangdong Provincial Key Laboratory of Marine Biotechnology, Shantou University, Shantou, Guangdong, China; 2 STU-UMT Joint Shellfish Research Laboratory, Shantou University, Shantou, Guangdong, China; 3 Institute of Tropical Aquaculture and Fisheries, Universiti Malaysia Terengganu, Kuala Terengganu, Terengganu, Malaysia; 4 Aquaculture Department, Southeast Asian Fisheries Development Center, Iloilo, Philippines; 5 Division of Biological Sciences, College of Arts and Sciences, University of the Philippines Visayas, Ioilo, Philippines; 6 Faculty of Marine Science and Fishery, Hasanuddin University, Makassar, Indonesia; 7 Faculty of Resource Science and Technology, Universiti Malaysia Sarawak, Kota Samarahan, Sarawak, Malaysia; 8 School of Fisheries and Aquaculture Sciences, Universiti Malaysia Terengganu, Kuala Terengganu, Terengganu, Malaysia

**Keywords:** *Scylla*, Discriminant Function Analysis, Morphometric, Presumed Hybrids, Mud Crab

## Abstract

There are four species of mud crabs within the genus *Scylla*, and most of them live sympatrically in the equatorial region. Apart from a report in Japan about the finding of a natural *Scylla* hybrid more than a decade ago after the division of genus *Scylla* into four species by Keenan, Davie & Mann (1998), no subsequent sighting was found. Thus, this study investigates the possible natural occurrence of potential hybridization among *Scylla* species in the wild. A total of 76,211 individuals from mud crab landing sites around the Malacca Straits, South China Sea and Sulu Sea were screened. In addition to the four-purebred species, four groups (SH 1, *n* = 2, 627; SH 2, *n* = 136; SH 3, *n* = 1; SH 4, *n* = 2) with intermediate characteristics were found, mostly at Sulu Sea. Discriminant Function Analysis revealed that all* Scylla* species, including SH 1 - 4, are distinguishable via their morphometric ratios. The most powerful discriminant ratios for each character and the top five discriminant ratios of males and females were suggested. The carapace width of SH 1 males and females were significantly smaller than pure species. Based on the discriminant ratios and the description of morphological characters, we hypothesize that the additional four groups of *Scylla* with intermediate characteristics could be presumed hybrids. Future work at the molecular level is urgently needed to validate this postulate.

## Introduction

Mud crabs belonging to the genus *Scylla* are distributed along the Indo-West-Pacific region and are divided into four distinct species, namely *S. serrata, S. olivacea, S. tranquebarica* and *S. paramamosain* (Keenan, Davie & Mann, 1998). All four species are economically important and highly sought after due to their delicate meat, making them important resources in both artisanal fisheries and aquaculture sector around the Indo-West-Pacific region (Waiho et al., 2018; Shi et al., 2019), with a global capture production of more than 20,000 t and aquaculture production of more than 100,000 t within the last decade (FAO, 2018).

The detailed morphological and morphometric differences among the four species in the genus *Scylla*—*S. serrata, S. tranquebarica, S. paramamosain, S. olivacea*—were described by Keenan, Davie & Mann (1998) and were used as the basis for species differentiation in most studies on mud crabs (Klinbunga, Boonyapakdee & Pratoomchat, 2000; Imai et al., 2004; Ikhwanuddin et al., 2011; Fazhan, Waiho & Ikhwanuddin, 2017b; Waiho et al., 2017b; Waiho et al., 2017c; Waiho et al., 2018), including the current study. These morphological differences are also used by farmers and crabbers to differentiate among species. Apart from morphological variations, variation in body size is also obvious among *Scylla* species. *S. serrata* is the largest, followed by *S. tranquebarica* and *S. paramamosain* (Ogawa et al., 2012; Fazhan et al., 2017a). *S. olivacea* is considered as the smallest among the four species (Waiho, Fazhan & Ikhwanuddin, 2016b; Fazhan et al., 2017a). However, due to the acclimatization to their natural habitat (upper river mouths with lower salinity and constant exposure to intertidal currents; (Fazhan et al., 2017a), *S. olivacea* are the sturdiest.

Inter-specific hybridization—the crossing of two different species—has been a common approach in the aquaculture sector for genetic improvement of aquaculture species, especially among finfishes (Bartley, Rena & Immink, 2000). The offspring, known as hybrids, may have some or combined desirable characteristics of both parents (positive heterosis), which is the ultimate goal of hybridization. Some of the successful inter-specific hybridization examples include hybrid striped bass (*Morone chrysops* × *M. saxatilis*) with faster growth, better temperature tolerance, flexibility to environmental parameters, and stronger disease resistance compared to its parents (Kohler, 2004; Ende et al., 2018), and Splake, hybrid trout (*Salvelinus namaycush* × *S. fontinalis*) that is fertile, fast growth and tolerant to acid water (Snucins, 1993).

Inter-specific hybridization occurs widely in fishes under natural conditions (Hubbs, 1955; Kirkpatrick, Everitt & Evans, 2007; Adams et al., 2014), thus explaining their higher feasibility of induced hybridization among fish species. Natural hybridization is less commonly reported in crustaceans, especially those in the infraorder Brachyura. Among the reported naturally hybridized species includes between red snow crab (*Chionoecetes japonicus*) and snow crab (*C. opilio*) in Korea (Kim et al., 2012) and between Intertidal stone crabs, *Menippe mercenaria* and *M. adina* in the Mexico Gulf (Wilber, 1989). Both snow crabs and intertidal stone crabs exhibited overlapping range, thus promoting the formation of natural hybrids (Wilber, 1989; Kim et al., 2012).

It is widely accepted that only three species of mud crabs (*S. olivacea, S. paramamosain* and *S. tranquebarica*) exist sympatrically in Malaysian waters (Ikhwanuddin et al., 2011; Waiho et al., 2016a; Waiho, Fazhan & Ikhwanuddin, 2016b; Fazhan, Waiho & Ikhwanuddin, 2017b). Additionally, some mud crabs with ambiguous characters were unexpectedly found among crab samples in the equatorial region during previous study (Fazhan et al., 2017a). Previously, Imai & Takeda (2005) reported the first occurrence of natural hybrid of mud crab in Japan—fathered by *S. serrata* with a female *S. olivacea*. This shows that natural hybridization in *Scylla* is possible in the natural environment. The potential of hybridization among *Scylla* in captivity has also been proven in a previous study based on their mating successes (Fazhan et al., 2017c). Males of *S. olivacea* were the most versatile and readily choose females of other species when *S. olivacea* females were not available, with inter-species mating percentage as high as that of pure species.

Therefore, this study aimed to address the feasibility of inter-specific natural hybridization among *Scylla* species by screening for crabs with ambiguous characteristics at known overlapping regions—the Malacca Straits, South China Sea and Sulu Sea. In addition, due to the small sample size used by Keenan, Davie & Mann (1998) during their description of genus *Scylla*, the current study built upon and strengthened Keenan et al.’s descriptions (both morphological and morphometric descriptions) with the inclusion of additional groups that possess minor variations in their morphological characters compared to the pure species. Further, a list of significant discriminant morphometric ratios capable of distinguishing pure species and SH 1–4 was also provided. This would be helpful to aquaculturists and researchers in species identification and selection. Lastly, the heterosis of SH 1 was highlighted based on the morphometric ratios, providing valuable information to the future selection of inter-specific hybridization candidate of *Scylla* species.

## Materials & Methods

### Sample collection

Mud crabs were screened from fishermen’s landings for a period of three years—September 2012 to August 2015. Sampling locations covered most mud crab landing sites representing the Malacca Straits, South China Sea and Sulu Sea. All sampling sites are common crab landing grounds and no licensing is required for the acquisition of mud crabs. None of the work involved endangered or protected species. Only sexually mature crabs with CW above 95 mm were measured to avoid samples of juvenile crabs that may yet to undergo ontogenic changes as they develop to become adults (Keenan, Davie & Mann, 1998; Waiho et al., 2016a; Waiho, Fazhan & Ikhwanuddin, 2016b). The total number of the crab screened was 76,211 individuals (Table S1). They were identified up to the species level based on the morphological keys described by Keenan, Davie & Mann (1998). A total of 1,524 *S. olivacea*, 1,399 *S. tranquebarica* and 1,441 *S. paramamosain* were randomly selected for CW measurement.

### Morphological description

The morphological features of all hybrids and pure species (*S. olivacea, S. tranquebarica, S. paramamosain* and *S. serrata*) were described. The key features were: (i) colouration, (ii) patterning on chelipeds, (iii) patterning on walking legs, (iv) shape and height of frontal lobe spines, (v) shape of carpus spine of chelipeds, and (vi) shape of propodus spine of chelipeds as depicted in Fig. S1.

### Morphometric measurement

Twenty-four morphometric characters (modified from Keenan, Davie & Mann, 1998) of mud crabs—pure species and *Scylla* groups with ambiguous morphological characteristics (SH) (300 males and 300 females for each species, or the highest number available)—were measured using standard Vernier caliper to the nearest 0.01 mm. They were: carapace width (CW), CW at spine 8 (8CW), internal carapace width (ICW), carapace length (CL), posterior width of carapace (PWC), carapace frontal width (FW), frontal median spine height (FMSH), distance between frontal median spines (DFMS), distance between frontal lateral spines (DFLS), sternum width (SW), abdomen width (AW), third periopod merus length (3PML), third periopod carpus length (3PCL), fifth periopod dactyl width (5PW), fifth periopod dactyl length (5PL), dactyl length (DL), propodus depth (PD), outer propodus spine (OPS), inner propodus spine (IPS), inner carpus spine (ICS), outer carpus spine (OCS), propodus width (PW), propodus length (PL) and merus length (ML). In general, each of the 24 morphometric characters was used as divisor and formed ratios with the remaining 23 morphometric characters, thus resulting in a total of 552 ratios. All possible combination of 552 ratios obtained from the 24 measured morphometric characters were tested using Discriminant Function Analysis (DFA). The ratios of pure species (*n* = 300 for each species) were tested with the ratios of SH 1 male (*n* = 300) and female (*n* = 111), and SH 2 male (*n* = 136). *S. serrata* (*n* = 3), SH 3 (*n* = 1) and SH 4 (*n* = 2) were excluded in this test because of their low sample size.

### Data analysis

All data analyses were performed using Microsoft Excel 2016 and IBM SPSS Statistic ver. 20. Stepwise DFA was used to determine which ratios (discriminant variables) could discriminate among *Scylla* species. Minimum F value was set as 3.0 (represents 0.05 significance level). Two-way ANOVA with Welch’s correction (Welch, 1947) was used to compare the mean CW values of males and females of each species (Levene’s test: W_male_ = 22.03, W_female_ = 23.65, *P* < 0.001). Games Howell post-hoc test was used to detect differences among treatments. A significance level at *α* = 0.05 was applied in all statistical tests.

**Table 1:** Morphological description of *Scylla* species found in the present study.

Species	Frontal lobe spines		Cheliped		Polygonal patterning	Cheliped coloration
	Shape	Height^a^	Carpus spines	Propodus spines	
*S. serrata*	Blunt pointed	High in male, moderately high in female	Both spines obvious	Obvious	Present on all chelipeds and legs	Blue, purple or green
*S. tranquebarica*	Blunt	Moderately high in male, high in female	Both spines obvious	Obvious	Present on the third, fourth and fifth legs	Mostly purple, rarely green or blue
*S. paramamosain*	Triangular	High in both sexes	Inner spine absent, outer spine obvious	Obvious	Weak patterns only observed on the fifth leg	Green to bright yellow with black spot or stripe patterns unlike the polygonal pattern in *S. serrata*
64 *S. olivacea*	Rounded	Low in male, moderate in female	Inner spine absent, outer spine reduced	Reduced	Absent on all chelipeds and legs	Orange or red through brown to black
SH 1	Rounded	Moderate in both sexes	Inner spine absent, outer spine reduced	Reduced	Absent on all chelipeds and legs	Yellowish to dark brown with black stripe or spot like *S. paramamosain*
SH 2	Blunt or semi triangular	Moderately high	Both spines obvious	Obvious	Weak, absent or rarely observed with stronger patterns on the fifth leg	Green to dark purple, occasionally observed with black stripe pattern or spot on chelipeds like *S. paramamosain*
SH 3	Rounded or blunt	Moderate	Inner spine absent, outer spine obvious	Reduced	Absent on all chelipeds and leg	Orange and light to dark brown, occasionally observed with black stripe pattern or spot on chelipeds like *S. paramamosain*
SH 4	Blunt pointed	High	Inner spine absent, Outer spine obvious	Obvious	Strong polygonal pattern on cheliped, Weak polygonal pattern on the fifth leg	Yellowish with polygonal pattern like *S. serrata*

**Notes.**

a The classification of frontal lobe spine height was based on the values of mean frontal lobe height divided by frontal width measured between medial orbital sutures. High, 0.06; moderately high, 0.05; moderate, 0.04; low, 0.03 (modified from Keenan, Davie & Mann, 1998).

## Results

### Morphological differences among species of genus *Scylla*

**Figure 1:**
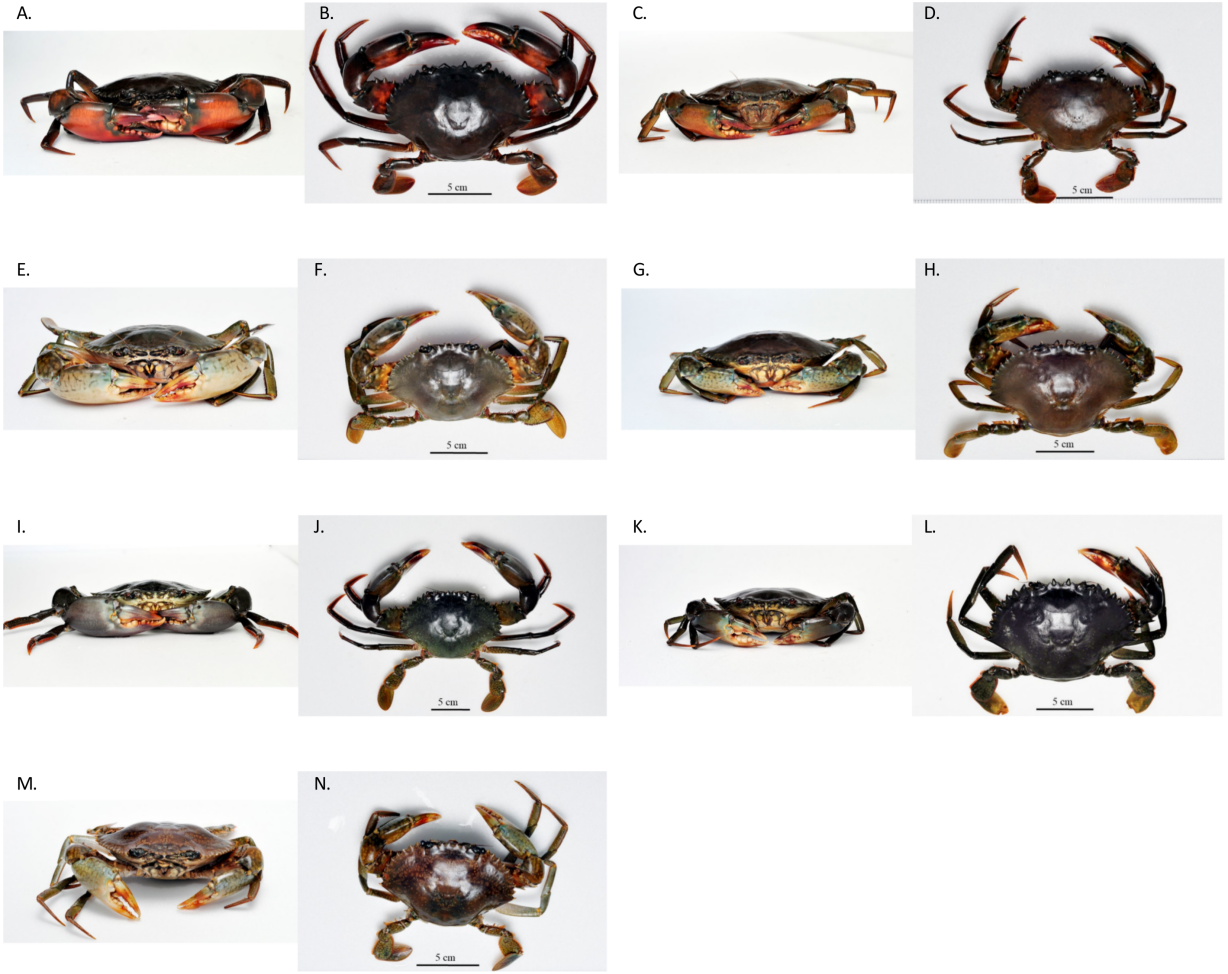
The frontal and dorsal view of pure *Scylla* spp. (A) and (B)—*S. olivacea* male; (C) and (D)—*S. olivacea* female; (E) and (F)—*S. paramamosain* male; (G) and (H)—*S. paramamosain* female; (I) and (J)—*S. tranquebarica* male; (K) and (L)—*S. tranquebarica* female; (M) and (N) –*S. serrata* female.

**Figure 2:**
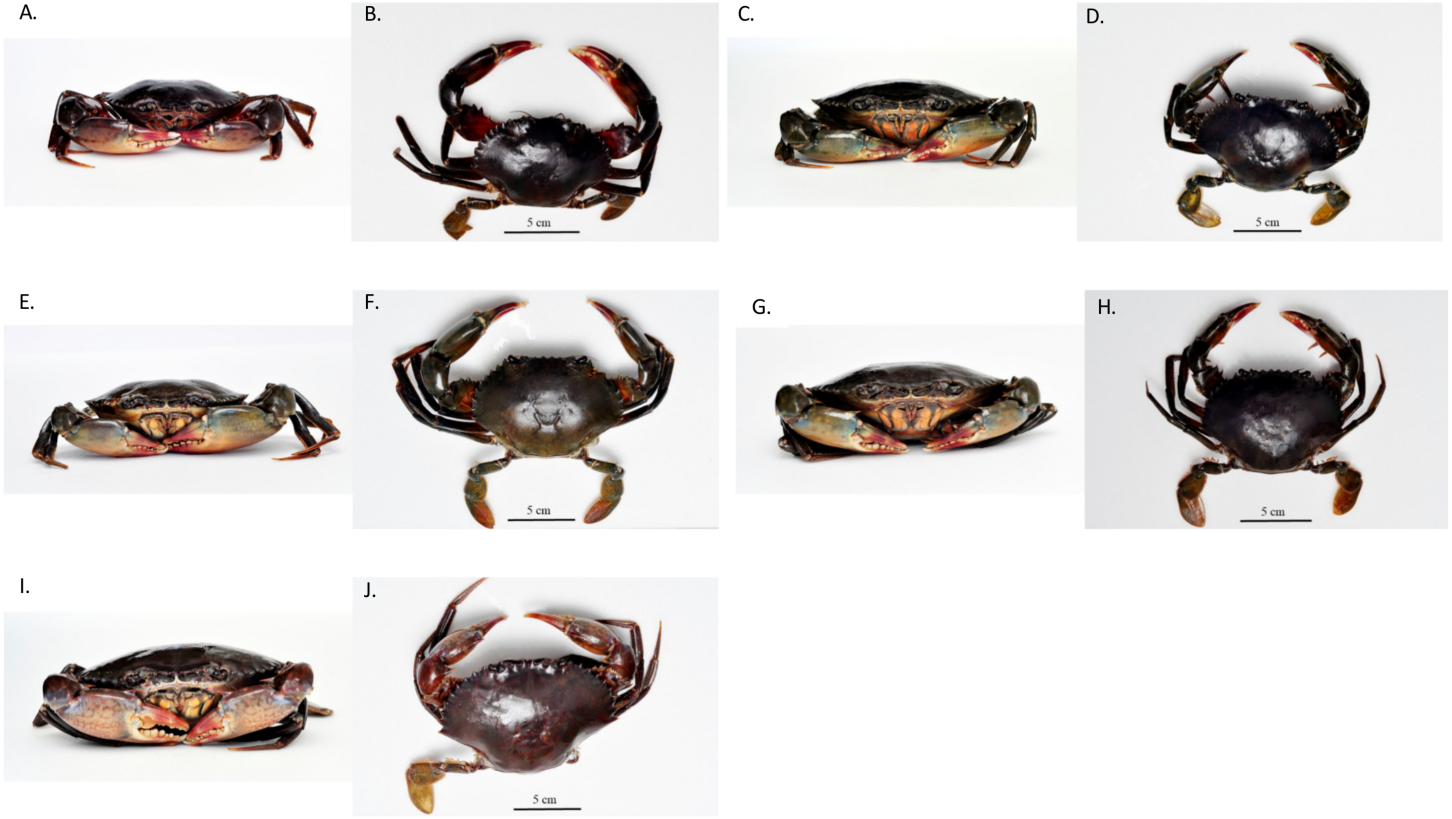
The frontal and dorsal view of additional *Scylla* groups. (A) and (B)—SH 1 male; (C) and (D)—SH 1 female; (E) and (F)—SH 2 male; (G) and (H)—SH 3 female; (I) and (J)—SH 4 female.

The four pure-species, *S. olivacea, S. paramamosain, S. tranquebarica* and *S. serrata* found in this study conformed with the morphological description provided by Keenan, Davie & Mann (1998). In addition, the external morphology of crabs that were ambiguous and deviates from Keenan’s classification were designated in different classes (SH 1–4) based on the characteristics that they accordingly possess (Table 1). The dorsal view and frontal view of male and female of each species, including SH 1–4 are shown in Figs. 1 and 2. SH 1–4 exhibited intermediate characteristics of the pure species. The morphological description of SH 1 is intermediate to that of *S. olivacea* and *S. paramamosain*, SH 2 is between *S. tranquebarica* and *S. paramamosain*, SH 3 is between *S. tranquebarica* and *S. olivacea*, and SH 4 is between *S. serrata* and *S. paramamosain*. All SH 1–4 were found only at Sulu Sea (Table S1).

### Morphometric differentiation

To provide a more complete coverage in addition to the ratios tested by Keenan, Davie & Mann (1998), a total of 552 ratios were analysed in this study. *Scylla serrata*, SH 3 and SH 4 were excluded from the analysis because of their low sample number. Each morphometric character, when used as a divisor and formed 23 ratios, was able to achieve a 100% discrimination among all tested groups (pure species and SH 1–2) (DFA, all *P* < 0.0001), except ratios divided by ICS. The most powerful discriminant ratios of top morphological characters for both sexes—≤ 5 discriminant ratios needed in males and females, and ≤12 discriminant ratios needed in males and females combined—as suggested by the DFA and listed in Table 2 (for range and average values see Table S2 for males and Table S3 for females; for the full list of the most powerful discriminant ratios generated from all morphometric character see Table S4; raw measurements can be found in Table S5) were able to distinguish among species up to 100%. For example, by using all five suggested discriminant ratios that are divided by FW, i.e., ICS/FW, FMSH/FW, 5PW/FW, OCS/FW and 3PCL/FW, males of pure species (*S. olivacea, S. tranquebarica* and *S. paramamosain*) and SH 1–2 may be distinguished. When both sexes were combined, at least 10–12 discriminant ratios based on ICW, FMSH, DL or IPS as divisors were needed to achieve 100% discrimination among all tested groups. It was also notable that when viewed collectively, more discriminant ratios were needed to reach a 100% discrimination among pure *Scylla* species and SH 1–2 (average of 14 discriminant ratios needed) compared to when species discrimination was conducted based on sex (average of 7 discriminant ratios in both males and females) (Table S4) (DFA, all *P* < 0.0001).

**Table 2:** Top morphometric characters with the most powerful discriminant ratios in *Scylla* spp.

	**Divisor**	**Number of discriminant ratios tested**	**Number of significant discriminant ratios (percentage of all combinations)**	**Number of insignificant discriminant ratios (excluded)**	**Excluded discriminant ratios**	**Most powerful discriminant ratios** **(number of ratios)**
***Male***						
	FW	24	22 (OG = 100%, CVG =100%)	2	FW/FW, ICW/FW, AW/FW	ICS/FW, FMSH/FW, 5PW/FW, OCS/FW, 3PCL/FW (5)
	FMSH	24	22 (OG = 100%, CVG =100%)	2	FMSH/FMSH, ICW/FMSH	ICS/FMSH, FW/FMSH, 5PW/FMSH, OCS/FMSH, 3PCL/FMSH (5)
	5PL	24	22 (OG = 100%, CVG =100%)	2	5PL/5PL, AW/5PL	ICS/5PL, FMSH/5PL, 3PCL/5PL, OCS/5PL, PD/5PL (5)
	5PW	24	21 (OG = 100%, CVG =100%)	3	5PW/5PW, ICW/5PW, AW/5PW	ICS/5PW, FMSH/5PW, 3PCL/5PW, OCS/5PW, FW/5PW (5)
	ML	24	21 (OG = 100%, CVG =100%)	3	ML/ML, ICW/ML, AW/ML	ICS/ML, FMSH/ML, 3PCL/ML, 5PL/ML, OCS/ML (5)
	PW	24	22 (OG = 100%, CVG =100%)	2	PW/PW, AW/PW	ICS/PW, FMSH/PW, 3PCL/PW, 5PL/PW, OCS/PW (5)
***Female***						
	8CW	24	20 (OG = 100%, CVG =100%)	4	8CW/8CW, DFLS/8CW, SW/8CW, DL/8CW	ICS/8CW, OPS/8CW, 3PCL/8CW, IPS/8CW, 5PL/8CW (5)
	PWC	24	17 (OG = 100%, CVG =100%)	7	PWC/PWC, DFMS/PWC, DFLS/PWC, FW/PWC, SW/PWC, DL/PWC, PD/PWC	ICS/PWC, OPS/PWC, 5PL/PWC, FMSH/PWC (4)
	CL	24	17 (OG = 100%, CVG =100%)	7	CL/CL, DFLS/CL, PD/DFLS, DL/DFLS, PL/DFLS, SW/DFLS, ML/DFLS	ICS/CL, FMSH/CL, OPS/CL, 3PCL/CL, IPS/CL (5)
	FMSH	24	18 (OG = 100%, CVG =100%)	6	FMSH/FMSH, SW/FMSH, PL/FMSH, DL/FMSH, DFLS/FMSH, ML/FMSH	ICS/FMSH, 5PL/FMSH, OPS/FMSH, PWC/FMSH (4)
	3PCL	24	19 (OG = 100%, CVG =100%)	5	3PCL/3PCL, PD/3PCL, DL/3PCL, SW/3PCL, DFLS/3PCL	ICS/3PCL, FW/3PCL, OPS/3PCL, IPS/3PCL, PW/3PCL (5)
	5PL	24	19 (OG = 100%, CVG =100%)	5	5PL/5PL, SW/5PL, DFLS/5PL, PD/5PL, DL/5PL	ICS/5PL, FMSH/5PL, OPS/5PL, 3PCL/5PL, IPS/5PL (5)
***Male + Female***						
	ICW	24	23 (OG = 100%, CVG =100%)	1	ICW/ICW	ICS/ICW, AW/ICW, FMSH/ICW, DL/ICW, 5PL/ICW, OCS/ICW, PD/ICW, 3PCL/ICW, IPS/ICW, CL/ICW, PW/ICW, OPS/ICW (12)
	FMSH	24	23 (OG = 100%, CVG =100%)	1	FMSH/FMSH	ICS/FMSH, AW/FMSH, PL/FMSH, 3PML/FMSH, OCS/FMSH, 5PL/FMSH, IPS/FMSH, 3PCL/FMSH, ICW/FMSH, OPS/FMSH, PD/FMSH, DL/FMSH (12)
	DL	24	23 (OG = 100%, CVG =100%)	1	DL/DL	ICS/DL, AW/DL, FMSH/DL, 5PL/DL, PD/DL, CL/DL, OCS/DL, 3PCL/DL, IPS/DL, CW/DL, PW/DL, OPS/DL (12)
	IPS	24	23 (OG = 100%, CVG =100%)	1	IPS/IPS	ICS/IPS, PW/IPS, AW/IPS, OCS/IPS, 3PCL/IPS, FMSH/IPS, CW/IPS, 3PML/IPS, 5PL/IPS, PD/IPS (10)

**Notes.**

OG original grouped cases correctly classified CVG cross-validated grouped cases correctly classified CW carapace width ICW internal carapace width 8CW CW at spine 8 PWC posterior width of carapace CL carapace length FW frontal width FMSH frontal median spine height DFLS distance between frontal lateral spines DFMS distance between frontal median spines SW sternum width AW abdomen width 3PML third periopod merus length 3PCL third periopod carpus length 5PL fifth periopod dactyl length 5PW fifth periopod dactyl width DL dactyl length PD propodus depth PL propodus length ML merus length PW propodus width IPS inner propodus spine OPS outer propodus spine OCS outer carpus spine ICS inner carpus spine

**Figure 3:**
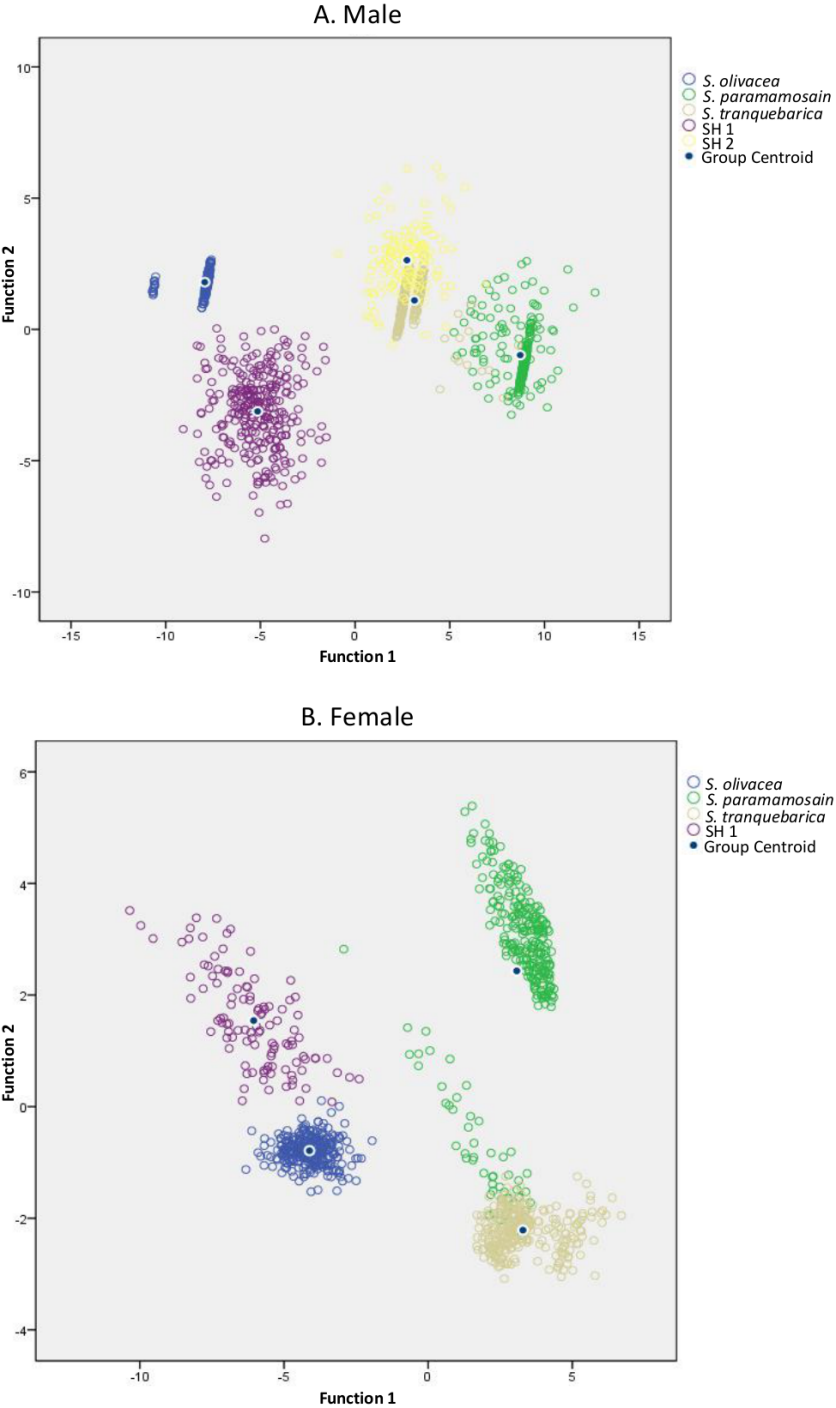
Canonical discriminant function graphs of *Scylla* species based on the top five selected ratios. (A)—male had a 97.1% confidence; (B)—female had a 93.2% confidence. The overall top five selected ratios were FMSH/ICW, FMSH/FW, FMSH/SW, FMSH/3PCL and FMSH/PL for males, and IPS/ICW, FMSH/FW, 3PCL/AW, FMSH/5PW and IPS/OPS for females, respectively.

In addition, to facilitate easier screening, the top five discriminant ratios with the ability to discriminate the species up to 100% in male and female were selected, one from each body parts, i.e., carapace, frontal lobe, abdomen, periopod and cheliped (Table 3). These discriminant ratios were able to distinguish among *Scylla* species, including SH 1–2, with 97.1% (DFA, *F* = 8, 442.13, *P* < 0.0001, Wilk’s Λ = 0.002) confidence in males (Fig. 3A) and 93.2% (DFA, *F* = 4932.58, *P* < 0.0001, Wilk’s Λ = 0.007) confidence in females (Fig. 3B). However, individually, each ratio showed less efficiency in discriminating among species, i.e., when used individually in males, the ratio of FMSH/PL, FMSH/ICW, FMSH/3PCL, FMSH/SW and FMSH/FW showed only 62.8%, 72.2%, 84.0%, 85.6% and 88.7% discrimination, respectively; in females, the ratio of 3PCL/AW, FMSH/5PW, FMSH/FW, IPS/OPS and IPS/ICW accounted for only 62.8%, 75.5%, 77.3%, 81.4% and 83.8% discrimination, respectively (DFA, all *P* < 0.0001). Thus, it is recommended to use these ratios in combination.

**Table 3:** The top five discriminant ratios in male (marked in bold) and female *Scylla* species.

Ratio	*S. olivacea* (*n*_male_ = 300; *n*_female_ = 300)		*S. tranquebarica* (*n*_male_ = 300; *n*_female_ = 300)		*S. paramamosain* (*n*_male_ = 300; *n*_female_ = 300)		SH 1 (*n*_male_ = 300; *n*_female_ = 111)		SH 2 (*n*_male_ = 136)	
	Mean ± sd	Range	Mean ± sd	Range	Mean ± sd	Range	Mean ± sd	Range	Mean ± sd	Range
**FMSH/ICW**	**0.0156 ± 0.00074**	**0.01305–0.01726**	**0.0210 ± 0.00118**	**0.01827–0.02489**	**0.0248 ± 0.00103**	**0.02240–0.02857**	**0.0162 ± 0.0109**	**0.01320–0.01917**	**0.0230 ± 0.00121**	**0.02037–0.02670**
**FMSH/FW**	**0.0324 ± 0.00099**	**0.02789–0.03275**	**0.0520 ± 0.00163**	**0.05115–0.06392**	**0.0634 ± 0.00190**	**0.0553–0.07157**	**0.0383 ± 0.00290**	**0.03034–0.04680**	**0.0499 ± 0.00253**	**0.04356–0.05783**
**FMSH/SW**	**0.0264 ± 0.00080**	**0.02275–0.0267**	**0.0391 ± 0.00114**	**0.03814–0.04684**	**0.0472 ± 0.00134**	**0.04230–0.05156**	**0.0303 ± 0.00236**	**0.02041–0.03701**	**0.0406 ± 0.00195**	**0.03628–0.04717**
**FMSH/3PCL**	**0.0517 ± 0.00157**	**0.04447–0.05222**	**0.0821 ± 0.00343**	**0.07870–0.10005**	**0.0988 ± 0.00324**	**0.08503–0.10959**	**0.0715 ± 0.00571**	**0.05715–0.08989**	**0.0795 ± 0.00389**	**0.06970–0.09130**
**FMSH/PL**	**0.0180 ± 0.00055**	**0.01547–0.01817**	**0.0300 ± 0.00261**	**0.02699–0.03246**	**0.0317 ± 0.00086**	**0.02838–0.03495**	**0.0203 ± 0.00151**	**0.01633–0.02487**	**0.0287 ± 0.00152**	**0.02455–0.03428**
IPS/ICW	0.0149 ± 0.00024	0.01437–0.01554	0.0301 ± 0.00396	0.02340–0.03839	0.1697 ± 0.06025	0.03031–0.23308	0.0317 ± 0.00549	0.02286–0.04141	–	–
FMSH/FW	0.0384 ± 0.00331	0.02948–0.04901	0.0599 ± 0.00458	0.05321–0.07198	0.0654 ± 0.00417	0.05156–0.06741	0.0382 ± 0.00164	0.03233–0.04991	–	–
3PCL/AW	0.6061 ± 0.05025	0.38871–0.62393	0.6460 ± 0.05635	0.49942–0.75567	0.6562 ± 0.05221	0.44869–0.77034	0.4870 ± 0.02221	0.43199–0.54036	–	–
FMSH/5PW	0.0959 ± 0.00731	0.07732–0.12151	0.1436 ± 0.01307	0.12124–0.18473	0.1508 ± 0.00429	0.13271–0.15808	0.1009 ± 0.01039	0.08122–0.12418	–	–
IPS/OPS	3.8121 ± 0.10768	3.57895–4.02941	1.1088 ± 0.22203	0.69697–1.67188	4.1584 ± 1.28949	0.79942–8.07576	1.3011 ± 0.34061	0.72222–2.16071	–	–

**Notes.**

n number of individual FMSH frontal median spine height ICW internal carapace width FW frontal width SW sternum width 3PCL third periopod carpus length PL propodus length IPS inner propodus spine AW abdomen width 5PW fifth periopod dactyl width OPS outer propodus spine

### Negative heterosis

Body size (CW) is among the most important economic trait in aquaculture production. The CW of male and female of all species (Table S6) were compared to determine if heterosis occurred in SH 1. Significant differences were observed in the CW of males (ANOVA, *F*_4,794.5_ = 18.47, *P* < 0.0001) and females (ANOVA, *F*_3,541.1_ = 24.17, *P* < 0.0001) among *Scylla* species. In males, SH 1 was significantly smaller compared with analysed *Scylla* species (*S. olivacea, S. tranquebarica, S. paramamosain* and SH 2) (Tukey Test: all *P* < 0.0001) whereas SH 2 was significantly larger than SH 1 (Tukey Test: *P* < 0.0001) but showed no difference in CW when compared with *S. olivacea, S. tranquebarica* and *S. paramamosain* (Games Howell, all *P* >0.09). Similar pattern was also observed in the CW of SH 1 females, in which the CW of females of all pure species (*S. olivacea, S. tranquebarica* and *S. paramamosain*) were significantly larger than the CW of SH 1 females (Games Howell, all *P* <  0.01).

## Discussion

### Morphological and morphometric characterization of *Scylla*

The combination of morphological and morphometric analyses in assessing species identity, especially in *Scylla* species was previously carried out by Keenan, Davie & Mann (1998). Present study confirmed that the morphological characters of the four pure *Scylla* species (*S. olivacea, S. paramamosain, S. tranquebarica* and *S. serrata*) described by Keenan, Davie & Mann (1998) was similar to the morphological characteristics of all four pure-species found in this study. Furthermore, the morphological characters of SH 1–4 (Table 1) described in our study would serve as a guideline for the identification and selection of mud crabs, and the characterization of hybrids in future inter-specific hybridization trials.

The selected discriminant ratios for each morphological character as described in Tables S2 and S3 confirmed the differences among *Scylla* species and the newly described SH 1–2 in terms of morphometric measurements. Among all the morphological characters measured, only *S. serrata* and *S. tranquebarica* exhibit measurable ICS whereas it is absent in *S. paramamosain, S. olivacea*, SH 1 (Table 3). Thus, as they are only useful in discerning *S. serrata* and *S. tranquebarica* from the other species, ratios using ICS as divisors are not recommended. Excluding ICS, other morphological characters, when each used as divisor, were able to distinguish among *Scylla* species, including SH 1–2, up to 100% with a minimum combination of ratios (Table 2). These are useful for future species identification, especially if only certain morphological characters are available for measurement.

The final selection of five ratios from different body parts in both sexes of *Scylla* allow easier reference for others in the future, with minimum morphometric characters to be measured (FMSH can be used as dividend for two ratios in females and all ratios in males). These five ratios (FMSH/ICW, FMSH/FW, FMSH/SW, FMSH/3PCL and FMSH/PL for males, and IPS/ICW, FMSH/FW, 3PCL/AW, FMSH/5PW and IPS/OPS for females) were also selected based on their ability to discriminate to above 93% confidence all species within genus *Scylla*, including SH 1–2 described in this study. In addition, these five discriminant ratios are measurements of common body parts that are noticeable and seldom worn out, unlike the ICS, OCS and CW, which may be reduced in intermoult crabs due to frequent abrasions with the surroundings. Of the suggested three most useful discriminant ratios to discriminate among *Scylla* species up to 94.1% by Keenan, Davie & Mann (1998), only two (FMSH/FW and ICS/OCS) matched those found in this study. These differences were observed because: (1) Keenan, Davie & Mann (1998) combined both sexes in their DFA, (2) only four *Scylla* species were explored and (3) their sample size was comparatively smaller (*n* = 68 for *S. serrata*, *n* = 25 for *S. tranquebarica*, *n* = 9 for *S. paramamosain* and *n* = 66 for *S. olivacea*). This study conducted DFA according to sex because some morphometric characters were reported to show sexual dimorphisms (Keenan, Davie & Mann, 1998). Thus, discriminant ratios based on sex would facilitate easier usage and differentiation of mud crabs for future researchers.

Although the number of crabs being classed as SH 3 and SH 4 were very low, their obvious morphological differences (Table 1) compared to others, i.e., *S. olivacea, S. serrata, S. tranquebarica, S. paramamosain*, SH 1 and SH 2, warrant them to be categorized into separate groups. The occurrence of hybrids, especially in crustaceans is not as common (Kim et al., 2012) as it is in other marine organisms such as fish (Bartley, Rena & Immink, 2000; Ende et al., 2018), and the report of one specimen of natural *Scylla* hybrid by Imai & Takeda (2005) in Japan further supports this postulate. However, it is important that the maternal and paternal inheritance of all four groups of SHs, including SH 3 and SH 4, should be tested using molecular methods to verify their hybrid status.

The characterization of purebred and those with ambiguous characters (SH 1–4), and the development of discriminant functions allow easier identification and selection of mud crab species for fisheries, aquaculture and research purposes. Correct species identification is crucial before incorporating any species into aquaculture. Our study provides the baseline morphometric data for future inter-specific hybridization trials of *Scylla* spp.

### Potential crosses in nature

The sympatric relationship between the three commonly found *Scylla* species (*S. olivacea, S. paramamosain* and *S. tranquebarica*) in Sulu Sea and their overlapping microhabitats highly increase their chances of encountering one another (Fazhan et al., 2017a), thus justifies the occurrence of SH 1, SH 2 and SH 3 found in this study. SH 4 (*n* = 2) was postulated to be the cross between *S. paramamosain* and *S. serrata*. This was highly surprising because prior to our study, *S. serrata* has never been reported in Malaysian waters. The discovery of three *S. serrata* therefore completely changed our current knowledge on the mud crab composition in the three seas representing Malaysian waters (Fazhan et al., 2017a; Fazhan, Waiho & Ikhwanuddin, 2017b). It was postulated that accidental release or escape was the main introduction vector as landing sites where *S. serrata* was found are known to import live *S. serrata* from neighbouring countries (Fazhan, Waiho & Ikhwanuddin, 2017b). SH 4 found in Sabah might have occurred due to the absence of natural population of *S. serrata* in the wild, forcing them to mate with another *Scylla* species sharing the same habitat, in this case with *S. paramamosain*.

Previously, Imai & Takeda (2005) reported only one hybrid specimen from Japan more than a decade ago and to date, no further sighting of natural hybrids was reported after that. The finding of large numbers of *Scylla* with ambiguous characters, especially SH 1 (*n* = 2,627, potential offspring of *S. olivacea* × *S. paramamosain*) around Sulu Sea is unprecedented. Based on our studies on the sampling location where SH 1 specimens were found, we postulate that one of the reasons could be due to the considerably large number of male and female *S. olivacea* (approximately 42% of the screened population) with abnormal sexual characters after infected by parasitic sacculinids (*Sacculina beauforti*) (Waiho et al., 2017a). These infected individuals, with reduced gonopods and pleopods, were sterile (Fazhan et al., 2018). With almost half of the population being infected, and the much lower species composition of *S. olivacea* (15%) compared to *S. paramamosain* (47%) (Fazhan et al., 2017a), healthy individuals might view other species as alternative mating partners. The feasibility of inter-species mating, especially *S. olivacea* from Sulu Sea in captivity has been reported in our previous study (Fazhan et al., 2017c). Future research on the mating preference of infected and non-infected *S. olivacea* with normal *S. paramamosain* could be useful to validate this postulate.

### Negative heterosis

The smaller body size (in terms of CW) of male and female SH 1 compared to its parents (male *S. olivacea* and female *S. paramamosain*) was observed in this study. SH 2 retained its parental growth trait (body size), with no significant difference in its CW with that of its parents (male *S. tranquebarica* and female *S. paramamosain*). Such negative heterosis or the absence of positive heterosis are not uncommon in inter-specific and intra-specific hybridization. For example, no notable heterosis in seven out of ten measured traits was reported in the hybrid of female *Clarias macrocephalus* and male *C. gariepinus* (Koolboon et al., 2014). Negative heterosis in terms of body length, body height and viability were observed in the intra-species cross of Chinese shrimp, *Fennropenaeus chinensis* from two different populations (Tian, Kong & Yang, 2006). The observations found in this study contribute greatly to the knowledge of natural hybridization in crustaceans and could serve as the basis for future studies on the induced hybridization of mud crab with desirable traits.

## Conclusions

The identity of the four *Scylla* species (*S. olivacea, S. tranquebarica, S. paramamosain* and *S. serrata*) was validated in this study. In addition, four additional groups (SH 1-4) that did not conform with the morphological description provided by Keenan, Davie & Mann (1998) were found. They (SH1-2) were also distinct compared to the four pure species based on their morphometric values analysed using DFA. This study also provides a list of comprehensive discriminant morphometric ratios based on each morphological character as divisors and suggests the best five ratios chosen from different body parts for males and females to distinguish among *Scylla* species, particularly to facilitate easier differentiation among pure *Scylla* species and the new-found groups. Future research on the molecular aspects of these groups is urgently needed to validate their potential hybrid status.

##  Supplemental Information

10.7717/peerj.8066/supp-1 Table S1 Screening numbers and locations of mud crab genus Scylla

10.7717/peerj.8066/supp-2 Table S2 The range and average values of the least needed ratios for each divisor (morphological character) to achieve a discriminant of 100% in males

10.7717/peerj.8066/supp-3 Table S3 The range and average values of the least needed ratios for each divisor (morphological character) to achieve a discriminant of 100% in females

10.7717/peerj.8066/supp-4 Table S4 Number of significant discriminant ratios and the most powerful discriminant ratios (100 % discrimination between group if combined) in males (S. olivacea, S. tranquebarica, S. paramamosain, SH 1, SH 2) and females (S. olivacea, S. tranquebarica, S. param

10.7717/peerj.8066/supp-5 Table S5 Raw measurements of the 24 morphometric characters of the genus Scylla

10.7717/peerj.8066/supp-6 Table S6 The mean carapace width, CW (mm) of male and female mud crab genus Scylla

10.7717/peerj.8066/supp-7 Figure S1 General anatomy and key features used in the identification of mud crab genus *Scylla*

## Additional Information and Declarations

### Data Availability

The following information was supplied regarding data availability: The raw measurements are available in the Supplemental Table.
