# Supplementary figures and images for: Morphological descriptions and morphometric discriminant function analysis reveal an additional four groups of Scylla spp
Source: PeerJ. 2020 Jan 3;8:e8066. doi: 10.7717/peerj.8066 (PMC6944125; doi:10.7717/peerj.8066)

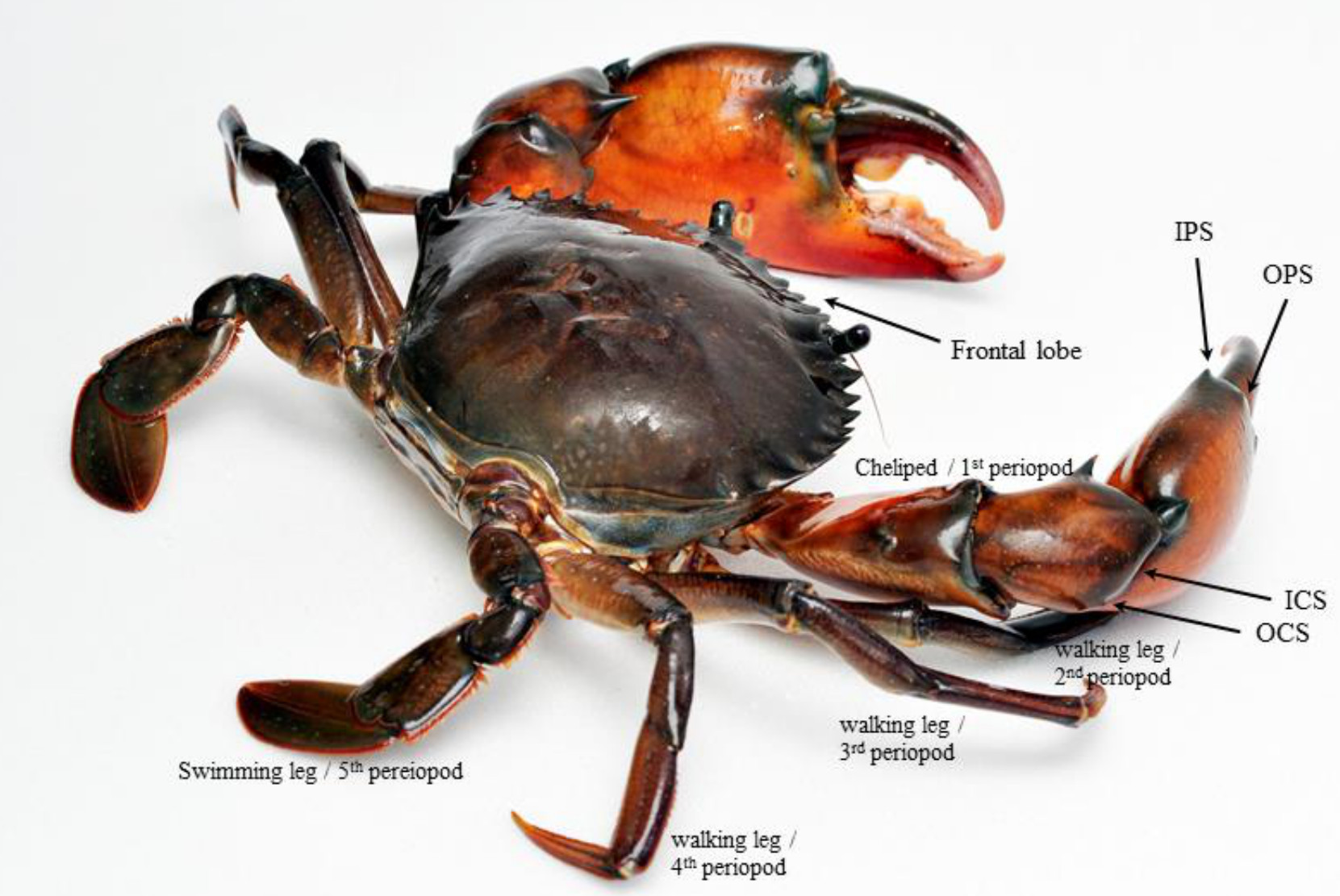

Supplement: Figure S1 [file peerj-08-8066-s007.jpeg]
